# M-GNN: A Topology-Enhanced Multi-Modal Graph Neural Network for Cancer Driver Gene Prediction

**DOI:** 10.3390/metabo16040268

**Published:** 2026-04-16

**Authors:** Lu Qin, Wen Zhu, Xinyi Liao, Yujing Zhang

**Affiliations:** 1 School of Mathematics and Statistics, Hainan Normal University, Haikou 571158, China; qluluq@yeah.net (L.Q.);; 2 Key Laboratory of Data Science and Intelligence Education, Hainan Normal University, Ministry of Education, Haikou 571158, China; 3 School of Mathematics and Systems Science, Guangdong Polytechnic Normal University, Guangzhou 510665, China; 4 China Unicom (Hainan) Industrial Internet Co., Ltd., Haikou 571924, China

**Keywords:** cancer driver genes, graph neural networks, multi-omics integration, topological feature enhancement, knowledge distillation

## Abstract

**Background:** Accurate identification of cancer driver genes is essential for understanding tumorigenesis and developing targeted therapies. Although graph neural networks (GNNs) have advanced multi-omics integration, existing methods often simply concatenate omics features and underutilize the topological information of biological networks. **Methods:** We propose M-GNN, a multi-modal GNN framework for cancer driver gene prediction. It employs separate Graph Convolutional Network (GCN) encoders to process four types of omics data (mutation, expression, methylation, copy number variation (CNV)), each represented as a 16-dimensional vector. We incorporate knowledge distillation by using soft labels from a pre-trained teacher model to enhance feature representation. An attention mechanism adaptively fuses the encoded omics features, and a dual-path classifier combining a GCN and a Multilayer Perceptron (MLP) preserves both intrinsic gene properties and network topology. **Results:** Experiments on three public protein–protein interaction (PPI) networks show that M-GNN consistently achieves the highest or second-highest AUPRC compared to five state-of-the-art methods. Ablation studies confirm the contribution of each module, and biological interpretability analysis—including analysis of GO enrichment and drug sensitivity—validates the reliability of the predicted genes. **Conclusions:** M-GNN provides a robust and interpretable computational tool for systematic cancer driver gene identification, effectively integrating multi-omics and network data.

## 1. Introduction

Cancer is a complex and highly lethal disease. According to the latest data from the National Cancer Center based on tumor registry and follow-up monitoring, the incidence of cancer in China in 2022 was about 4,824,700 new cases [1]. Studies have shown that all cancers are caused by mutations in somatic cells that accumulate and cause cancer [2]. Furthermore, cancer driver genes play a crucial role in the transformation of normal cells into cancerous cells, and therefore accurately predicting them is essential for effectively inhibiting tumorigenesis, advancing targeted therapies, and identifying rational drug targets [2,3].

Cancer, as a highly heterogeneous disease, involves complex changes across multiple levels. Research indicates that analyzing the interaction networks among genes can systematically investigate disease mechanisms and contribute to the identification of genes that drive cancer progression [4]. Conventional statistical approaches, such as MutSigCV [5] and OncodriveCLUST [6], rely on statistical models to pinpoint genes with unusually high mutation rates in cancer samples. However, these methods are limited by their inability to detect low-frequency driver genes and to accommodate sample heterogeneity. In contrast, methodologies like HotNet2 [7] and DawnRank [8] operate under the premise that driver genes occupy pivotal positions within molecular networks, where their mutations disrupt crucial cellular functional modules. These approaches leverage established biological networks (e.g., PPI networks) or construct co-expression networks. For instance, HotNet2 employs diffusion modeling to pinpoint network regions frequently mutated across multiple samples, while NetBox [9] integrates PPI networks and pathway data to establish a functional network and subsequently identify frequently mutated connectivity subnetworks. Traditional network approaches often rely on a single data type, which limits their ability to elucidate the mechanisms underlying cancer occurrence from multiple dimensions.

In recent years, GCNs have demonstrated the ability to uncover hidden driver signals and capture detailed domain context. Concurrently, accumulating evidence indicates that network topological features are crucial for identifying functionally important genes [10]. Consequently, numerous GNN-based deep learning approaches have emerged that integrate molecular networks and multi-omics data to predict cancer driver genes. EMOGI [11] is a pioneering GCN-based method that integrates multi-omics data with PPI networks using GCNs [12]. It constructs a graph where nodes represent genes (each with multi-omics features) and edges represent protein–protein interactions. Through multi-layer convolution, GCNs learn node embeddings by aggregating information from neighboring nodes, enabling the model to dynamically capture optimal feature representations from biological networks. This approach has proven effective in uncovering hidden driver signals and has inspired subsequent GNN-based methods for cancer gene prediction. HGDC [13], on the other hand, introduces graph diffusion (PPR) to generate auxiliary networks in three different biomolecular networks, namely, the pathway network, the RNA interaction network, and the PPI network, to adapt to the heterogeneous environment of the biomolecular networks. SGCD [14] uses representation separation (RS) to enhance the generalization ability of GNNs on heterophilic graphs, alleviating the feature confusion problem that traditional GNNs tend to cause when aggregating node information in heterogeneous networks. These methods usually construct the graph by directly splicing multi-omics features together with PPI networks or other multi-networks such as gene co-expression and pathway co-occurrence, without accounting for variability across different omics. Multi-view representation learning techniques have been explored for fusing multi-omics data. Yang et al. proposed a multi-omics data fusion method based on multi-view GNN-MVGNN [15] for breast cancer classification; however, this approach does not consider the structural relationships of genes within the PPI network. In contrast, deepCDG [16] employs a shared GCN model to aggregate features from specific omics data and subsequently applies attention mechanisms for data fusion. IMVRL-GCN [17] designs shared and specific representation learners to capture shared and specific representations from multi-view data. Although this approach effectively models the complex dependencies among multiple source features, it fails to adequately account for the differences and deep interactions between various omics, nor does it efficiently integrate multi-perspective data.

Transfer learning and pre-training models hold significant promise for improving the prediction of cancer driver genes [18]. Notable advancements have been made in this area, exemplified by TMO-Net [19], a tumor multi-omics pre-training network that integrates diverse multi-omics pan-cancer datasets. This integration facilitates model pre-training, enabling adaptation to various downstream tasks in oncology and establishing a crucial methodology for future multi-omics cancer research. Geneformer [20], an attention-based deep learning model designed for context awareness, demonstrates that fine-tuning downstream tasks with limited task-specific data following pre-training on a large-scale corpus enhances prediction accuracy.

Knowledge distillation (KD) has emerged as a complementary technique to transfer learning, where a larger teacher model guides a compact student model by providing soft labels, improving generalization and stability [21]. KD has been successfully applied in GCNs and bioinformatics, demonstrating its effectiveness in distilling structural and semantic knowledge [22,23,24]. Inspired by these advances, we adopt a knowledge distillation strategy in this work, where a teacher model pre-trained on the full dataset provides soft labels to guide the student model’s training, thereby enhancing feature representation and generalization.

**Figure 1:**
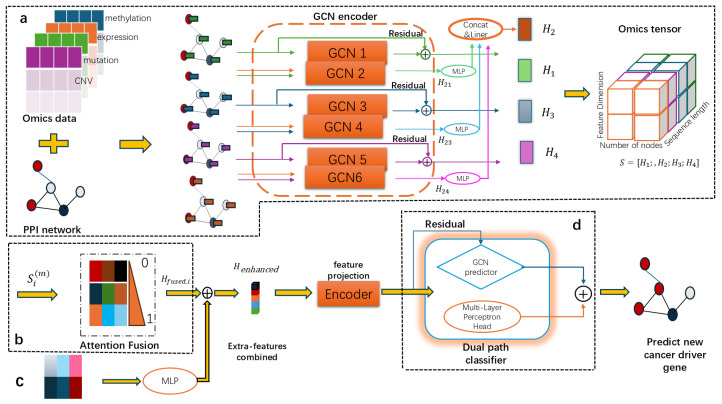
Overview of the proposed M-GNN framework. (**a**) Multi-omics data (mutation, expression, methylation, CNV) are encoded by separate GCN branches with residual connections. Expression data are further processed through three interaction encoders to capture cross-omics patterns. (**b**) The cross-group interaction fusion layer employs an attention mechanism to adaptively integrate the four omics embeddings. (**c**) The feature enhancement module incorporates additional features: PPI structural features, Node2Vec embeddings, and teacher model logits via knowledge distillation. (**d**) The dual prediction head consists of a GCN-based path (capturing network topology) and an MLP-based path (capturing node-intrinsic features), whose outputs are summed to produce the final prediction. “⊕” indicates summation, and “linear” refers to the linear layer.

In this work, we present a framework for predicting cancer driver genes that integrates multi-omics data using a multi-modal GCN (M-GNN). As illustrated in Figure 1, the M-GNN framework consists of four core modules: a multi-omics encoder, a cross-group interaction fusion layer, a feature enhancement module, and a dual prediction head. The core innovation of this framework lies in its ability to achieve data integration and feature learning at multiple levels and granularities. Initially, we enhance the original features by incorporating topological features derived from PPI networks to capture the significance of genes from diverse perspectives. Additionally, Node2Vec embedding is employed to learn distributed gene representations within the network, capturing intricate functional similarities through unsupervised pre-training. Furthermore, we enhance model performance by training a teacher model independently on the complete training set with optimized configurations. The distilled knowledge from the teacher model is utilized as supplementary node features, and a distillation loss is introduced to further enhance the model’s predictive capabilities. Specialized multi-encoder GCN structures are developed to independently process distinct omics. For expression data, a multi-view learning approach is utilized to capture interaction patterns with other omics using three distinct interaction encoders. A dual-path classifier design is implemented, merging the advantages of GNN and MLP paths to enable the model to leverage network topology information while not being overly dependent on network connectivity quality. Empirical findings on the pan-cancer dataset underscore the robustness and predictive efficacy of the proposed model.

## 2. Materials and Methods

### 2.1. Materials

Our multi-omics data were obtained from the Cancer Genome Atlas (TCGA) repository (https://portal.gdc.cancer.gov/, accessed on 13 May 2025), encompassing tumor genomic, epigenomics, and transcriptomics data from more than 29,446 samples of 16 different malignancies. For each cancer type, we calculated four biological characteristic metrics for each gene: gene mutation rate, copy number alteration rate, differential DNA methylation rate, and differential expression rate. Specifically:

- **Gene mutation rate:** The average number of non-silent mutations in the gene divided by its exonic length across all tumor samples of that cancer type.
- **Copy number alteration rate:** The proportion of samples in which the gene exhibits amplification or deletion within that cancer type.
- **Differential DNA methylation rate:** The average difference in beta values between tumor and paired normal samples for CpG sites within the gene region.
- **Differential gene expression rate:** The average log2 fold change of expression levels between tumor and paired normal samples, after filtering out genes with zero values in more than 10% of samples and log2 transformation.

By concatenating these vectors across all 16 cancer types, we constructed a 64-dimensional feature vector for each gene, with each omics contributing 16 dimensions. Subsequently, we performed feature standardization (Z-score normalization) on the 64-dimensional feature vectors. To avoid data leakage, the mean and standard deviation for each feature were calculated solely on the training set within each cross-validation fold and then applied to standardize the corresponding validation and test sets. This ensured that no information from the validation or test sets influenced the normalization parameters, which would otherwise constitute data leakage and lead to overly optimistic performance estimates. All features were normalized to have zero mean and unit variance based on the training fold statistics (for further details, please refer to Section S2 of the Supplementary Materials).

The PPI networks were compiled from three public databases:STRINGdb [25], CPDB [26], and IRefIndex [27]. STRINGdb employs a threshold of 0.85, while the CPDB network includes only interactions with confidence scores exceeding 0.5. The IRefIndex considers only binary human protein interactions. In this study, our PPIs served not only as input for the encoder but also for the extraction of structural features. To ensure consistency in the format of the PPI data, we standardized gene names from various formats into uniform symbolic names. Each gene is represented as a node in the graph, with edges constructed between nodes to illustrate the corresponding protein–protein interactions. Consequently, we obtained a total of three PPIs in a unified format, and an overview of these PPIs is presented in Table S1 of the Supplementary Materials. For each network, the numbers of positive samples (known driver genes) and negative samples (remaining genes) were as follows: STRINGdb: 783 positive (5.94%) and 12,396 negative; CPDB: 796 positive (5.84%) and 12,831 negative; IRefIndex: 836 positive (4.91%) and 16,177 negative. These numbers reflect the severe class imbalance inherent to the driver gene prediction task.

To ensure fair benchmarking comparisons, we utilized the same list of known driver genes as EMOGI [11], derived from the Network of Cancer Genes (NCG) v6.0 [28], the COSMIC Cancer Gene Census (CGC v91) [29], and DigSEE [30], which served as positive samples. Negative samples were obtained by excluding gene lists from the NCG, COSMIC, Mendelian Inheritance in Man and its online version (OMIM) [31], and the Kyoto Encyclopedia of Genes and Genomes (KEGG) cancer pathway [32]. This common strategy may inadvertently have included undiscovered driver genes in the negative set. To assess the potential impact of such a bias, we performed 10 independent repetitions of 5-fold cross-validation with different random seeds, which effectively generated different negative sample sets (while always excluding known drivers). The resulting standard deviation (Section 3.1) was low, indicating that our model’s performance was stable under different negative sample choices.

### 2.2. Methods

#### 2.2.1. Overview of M-GNN

As illustrated in Figure 1, M-GNN introduces a multi-level feature fusion architecture alongside a hybrid graph learning strategy. This model achieves precise predictions of cancer driver genes by systematically integrating fundamental genomic features, network topology features, and knowledge-enhanced features. It comprises four core modules: the multi-omics encoder, the cross-group fusion module, the feature-enhanced module, and the dual prediction header module. An overview of each module is provided below.

#### 2.2.2. Graph Structure Construction

We constructed a biomolecular network graph in which genes serve as nodes and PPIs function as edges. The graph is formally defined as G=(V,E,X), where *V* represents the set of gene nodes, *E* denotes the set of PPI edges, and $X\in R^{|V|\times 64}$ is the node feature matrix containing multi-omics features (16 dimensions each for mutation, expression, methylation, and CNV) associated with each gene.

Edge information was sourced from the PPI database, allowing us to construct the adjacency matrix *A*. To ensure the stability of the graph convolution operation, we applied symmetric normalization to the adjacency matrix with added self-loops. Specifically, we first added the identity matrix to the adjacency matrix to obtain $\hat{A}=A+I$. We then computed the degree matrix $\hat{D}$ of $\hat{A}$, where ${\hat{D}}_{ii}=\sum_{j}{\hat{A}}_{ij}$. The normalized adjacency matrix used in graph convolution was ${\hat{D}}^{-1/2}\hat{A}{\hat{D}}^{-1/2}$. This normalization balances the influence of high-degree and low-degree nodes and is widely adopted in GCNs [12].

#### 2.2.3. Multi-Omics Encoder

We developed an enhanced GCN encoder to learn low-dimensional representations of nodes within PPI networks. This encoder incorporates a residual connectivity mechanism, derived from the standard GCN, to improve gradient flow and model representation. To better capture the distinct characteristics of each grouping, we partitioned the 64-dimensional features into four 16-dimensional feature vectors. Each encoder processes a unique subset of features while maintaining a shared graph structure $\hat{A}$. Each encoder comprises two parallel pathways, a fully connected path and a graph convolution path, which merge their outputs through residual concatenation. This architecture enables the model to account for both a node’s intrinsic features and those of its neighboring nodes, thereby alleviating the issue of gradient vanishing in deep networks. To improve the robustness and generalization capability of the model, we introduced random perturbations to both the input data and the network structure during training. Specifically, we applied a random mask to the feature matrix *X* and randomly discarded edges from the adjacency matrix *A*, resulting in the enhanced feature matrix $X^{\prime }$ and the enhanced adjacency matrix $A^{\prime }$.

The formula can be stated as follows:

Let the input node features of layer *l* be $H^{\left(l\right)}\in R^{N\times d}$, where *N* represents the number of nodes and *d* denotes the feature dimension. The output of layer l+1 is computed as follows:

(1) $$\begin{array}{cc} H_{\mathrm{linear}}^{\left(l\right)} & =\sigma (H^{\left(l\right)}W_{L}^{\left(l\right)}) \end{array}$$

(2) $$\begin{array}{cc} H_{\mathrm{conv}}^{\left(l\right)} & =\sigma ({\hat{D}}^{\prime -\frac{1}{2}}\hat{A^{\prime }}{\hat{D}}^{\prime -\frac{1}{2}}H^{\left(l\right)}W_{c}^{\left(l\right)}) \end{array}$$

(3) $$\begin{array}{cc} H^{\left(l+1\right)} & =H_{\mathrm{linear}}^{\left(l\right)}+H_{\mathrm{conv}}^{\left(l\right)} \end{array}$$

where $\hat{A}=A+I$ is the adjacency matrix that incorporates self-connections, $\hat{D}$ is the degree matrix for $\hat{A}$, and $W_{L}^{\left(l\right)}$ and $W_{c}^{\left(l\right)}$ are the learnable weight matrices. Additionally, σ is the ReLU activation function. Furthermore, we applied Layer Normalization after the output of each encoder to stabilize the training process.

For multi-omics data, we employed distinct GCN encoders for mutation, methylation, and CNV to independently learn group-specific feature representations $H_{1}$, $H_{3}$, and $H_{4}$. To elucidate the interaction between expression omics and the other omics, we developed three specialized GCN encoders aimed at capturing the interaction patterns of expression omics with mutation, methylation, and CNV omics, respectively. Although these encoders share an identical network structure, they possess independent parameters, which facilitates the extraction of unique patterns related to different omics from the expression data. These patterns were subsequently processed by an MLP for each view, $H_{21}$, $H_{23}$, and $H_{24}$ (with independent parameters, i.e., no weight sharing), and were ultimately spliced and fused into a comprehensive representation of the expression omics $H_{2}$:

(4) $$\begin{array}{cc} H_{21}^{\prime } & ={\mathrm{MLP}}_{1}\left(H_{21}\right) \end{array}$$

(5) $$\begin{array}{cc} H_{23}^{\prime } & ={\mathrm{MLP}}_{2}\left(H_{23}\right) \end{array}$$

(6) $$\begin{array}{cc} H_{24}^{\prime } & ={\mathrm{MLP}}_{3}\left(H_{24}\right) \end{array}$$

(7) $$\begin{array}{cc} H_{2} & =\mathrm{Linear}(\mathrm{Concat}\left(H_{21}^{\prime },H_{23}^{\prime },H_{24}^{\prime }\right)) \end{array}$$

#### 2.2.4. Cross-Group Interaction Fusion Layer

We obtained four omics embedding representations $H_{1}$, $H_{2}$, $H_{3}$, and $H_{4}$, corresponding to mutation, fused expression, methylation, and CNV. Subsequently, we aimed to capture the higher-order interactions among these diverse omics data and perform feature fusion. To achieve this, we employed an attention fusion mechanism to integrate the multi-omics features. By computing attention weights for each omics embedding, the model adaptively learns the relative importance of different omics in predicting PPIs. This design significantly reduces complexity while preserving the capacity to interact with cross-omics information.

The four omics representations were initially stacked into a tensor $S=\left[H_{1};H_{2};H_{3};H_{4}\right]\in R^{4\times N\times d_{h}}$, where 4 represents the number of omics, *N* indicates the number of nodes, and $d_{h}$ signifies the hidden layer dimension. We subsequently calculated attention weights for each node in the omics dimension. For node *i*, we utilized matrix $S_{i}\in R^{4\times d_{h}}$, which encapsulates the embeddings of the four omics associated with that node. We then derived a score for each omics embedding at that node using the attention network, and then applied the softmax function to obtain the weights for node *i* and omics *m*, employing the specific formulas outlined below:

(8) $$a_{i}^{\left(m\right)}=\frac{\exp(\sigma \left(W_{a}S_{i}^{\left(m\right)}+b_{a}\right)v)}{\sum_{m=1}^{4}\exp(v^{T}\sigma \left(W_{a}S_{i}^{\left(m\right)}+b_{a}\right)}$$

where $W_{a}\in R^{d_{a}\times d_{h}}$ denotes the weight matrix of the attention mechanism, mapping the omics representation from $d_{h}$ to $d_{a}$ dimensions. $b_{a}\in R^{d_{a}}$ represents the bias term of the attention mechanism, while $v\in R^{d_{a}}$ is the output vector of the attention mechanism, which transforms the $d_{a}$-dimensional vector into a scalar. $a_{i}^{\left(m\right)}\in R^{N}$ indicates the attention weight vector for the *i*-th omics, with each element corresponding to the weight of a node.

Weighted fusion based on attentional weights:

(9) $$H_{\mathrm{fused},i}=\sum_{m=1}^{4}a_{i}^{\left(m\right)}⊙S_{i}^{\left(m\right)}$$

The fused representation $H_{\mathrm{fused},i}$ was utilized for final classification.

#### 2.2.5. Feature Enhancement Module and Dual Prediction Head Module

In the task of predicting cancer-driven genes, the high heterogeneity of cancers necessitates a prediction model with considerable flexibility [3]. The architecture of the dual prediction head is grounded in the theory of multi-perspective learning, which posits that analyzing data from various viewpoints results in a more comprehensive and robust representation [33].

##### Feature Enhancement Module

To fully leverage the topological information inherent in PPIs, we introduced two categories of unsupervised features, structural features and Node2Vec embeddings, along with soft-labeled teacher model logits derived from pre-trained teacher models through knowledge distillation. For the structural features, we computed seven metrics for each node [34,35,36,37]. The Node2Vec algorithm [38,39] was utilized to derive the distributed embedding representations of the nodes. Finally, we implemented a knowledge distillation framework [21] to enhance model performance. Specifically, we first trained a teacher model using the same multi-encoder architecture as M-GNN (MultiEncoderNet) but with increased capacity: it comprised 6 GCN layers, with hidden dimensions of 128 in the first classifier layer and 512 in the second classifier layer (which are sequential layers, i.e., the output of the first serves as the input to the second) (compared to 96 and 256 in the student). The teacher was trained on the complete training set (all nodes) for 1200 epochs using the same class-balanced binary cross-entropy loss as the student but without distillation. We employed an ensemble of 10 such teacher models (each initialized with different random seeds) and averaged their logits to produce soft labels. The hyperparameters were optimized via grid search on a validation set to maximize the AUPRC, resulting in a learning rate of 0.0003, a weight decay of 5 × 10^−5^, a dropout of 0.15, an early stopping patience of 200, and an exponential moving average decay of 0.995 (see Supplementary Materials Section S3.5 for full details). After training, the teacher generated soft labels (logits) for all nodes for subsequent use.

These soft labels were then used in two ways: (i) concatenated as additional input features to the student model (as shown in Equation (10)), and (ii) incorporated into a distillation loss (Equation (23)) that encouraged the student’s predictions to match the teacher’s soft labels. The overall loss combined task-specific binary cross-entropy with the distillation loss, weighted by a hyperparameter λ. The value of λ was set to 0.5 based on validation performance (explored within the range of 0.1 to 0.9 with a step of 0.1).

Detailed computational information and parameters are available in Section S3 of the Supplementary Materials. These features were integrated with the original multi-omics features to create an enhanced node feature representation.

(10) $$\begin{array}{cc} H_{\mathrm{extra}} & ={\mathrm{MLP}}_{\mathrm{extra}}\left(X_{\mathrm{extra}}\right) \end{array}$$

(11) $$\begin{array}{cc} H_{\mathrm{enhanced}} & =H_{\mathrm{fused},i}+H_{\mathrm{extra}} \end{array}$$

where $X_{\mathrm{extra}}$ denotes additional feature matrices such as Node2Vec embeddings or structural features.

##### Architectural Principle of Dual Predictive Head

To fully leverage the information from both the graph structure and the individual node features, we proposed a dual predictive head architecture. This architecture comprises a GCN-based predictive head for the graph structure and an MLP-based predictive head for node features. Prior to feeding the augmented representation into the predictive heads, we first adjusted its dimensionality using a projection layer. The same residual GCN architecture employed in the previously described omics encoder was utilized to project the representation into a 128-dimensional space.

**1. GCN Classifier Prediction Header.** In deep networks, residual connections facilitate the direct propagation of gradients through shortcuts, thereby enabling the training of deeper architectures [40]. We implemented residual connectivity in the GCN classifier prediction header to develop a deeper (6-layer) GCN, which mitigates gradient vanishing and model degradation, ultimately enhancing model representation. The propagation rule for each layer was as follows:

(12) $$\begin{array}{cc} H_{L,c}^{\left(l\right)} & =\mathrm{ReLU}(H^{\left(l\right)}W_{L,c}^{\left(l\right)}) \end{array}$$

(13) $$\begin{array}{cc} H_{c,c}^{\left(l\right)} & =\mathrm{ReLU}({\hat{D}}^{-\frac{1}{2}}\hat{A}{\hat{D}}^{-\frac{1}{2}}H^{\left(l\right)}W_{c,c}^{\left(l\right)}) \end{array}$$

(14) $$\begin{array}{cc} H^{\left(l+1\right)} & =\mathrm{Dropout}(H_{L,c}^{\left(l\right)}+H_{c,c}^{\left(l\right)},p_{\mathrm{dropout}}) \end{array}$$

where $H^{\left(l\right)}$ represents the node representation matrix of layer *l*, $W_{L,c}^{\left(l\right)}$ denotes the weight matrix for the linear transformation of layer *l*, and $W_{c,c}^{\left(l\right)}$ signifies the weight matrix for the graph convolution in layer *l*. The final output of the GCN classifier is given by

(15) $${\hat{y}}_{G}=H_{G}W_{G}+b_{G}$$

where $W_{G}$ is the weight matrix of the output layer and $b_{G}$ is the bias term for the output layer.

**2. MLP Forecast Header.** The MLP is a feed-forward neural network distinguished by its fully connected architecture, which enables the capture of node-intrinsic features. Consequently, the MLP predictor head is adept at focusing on these features [41]. The MLP executes a nonlinear transformation of the features via a fully connected layer, thereby learning the intricate mapping between node features and their corresponding labels.

The MLP prediction head uses a 3-layer feed-forward network structure specifically represented as follows:

(16) $$\begin{array}{cc} H_{m}^{\left(1\right)} & =\mathrm{ReLU}(\mathrm{LayerNorm}\left(H_{\mathrm{projected}}\right)W_{m,1}+b_{m,1}) \end{array}$$

(17) $$\begin{array}{cc} H_{m}^{\left(2\right)} & =\mathrm{ReLU}(H_{m}^{\left(1\right)}W_{m,2}+b_{m,2}) \end{array}$$

(18) $$\begin{array}{cc} H_{m}^{\left(3\right)} & =\mathrm{Dropout}(H_{m}^{\left(2\right)}W_{m,3}+b_{m,3},p_{\mathrm{dropout}}) \end{array}$$

The final output of the MLP predictor header is

(19) $${\hat{y}}_{m}=H_{m}^{\left(3\right)}W_{\mathrm{MLP}}+b_{\mathrm{MLP}}$$

where $W_{\mathrm{MLP}}$ denotes the output layer weight matrix, and $b_{\mathrm{MLP}}$ represents the output layer bias term.

##### Dual Prediction Head Fusion

The outputs of the two prediction heads are fused by summing:

(20) $$\hat{y}={\hat{y}}_{G}+{\hat{y}}_{m}$$

where $\hat{y}$ represents the final predicted logits. These logits are then converted to probabilities.

In binary classification tasks, the conversion is achieved using the sigmoid function:

(21) $$P\left(y_{i}=1\right)=\frac{1}{1+\exp(-{\hat{y}}_{i})}$$

### 2.3. Model Training

In cancer driver gene prediction, the extreme class imbalance (few positive samples) and high heterogeneity of cancer types pose significant challenges for model generalization. Knowledge distillation addresses these challenges by transferring dark knowledge from a high-capacity teacher model to the student, providing richer supervisory signals than one-hot labels and improving robustness to noisy features [21]. In this study, we employed a multi-component loss function to concurrently enhance model prediction accuracy and facilitate knowledge distillation. The loss function of our model comprises two components: task loss, represented by binary cross-entropy loss, and knowledge distillation loss, applicable when a teacher model is utilized. Furthermore, we incorporated class-balanced weights and provided the option of Focal Loss. A detailed explanation follows.

#### 2.3.1. Main Task Loss Function

To address the class imbalance issue in cancer driver gene prediction, we employed a class-balanced binary cross-entropy loss:

(22) $$L_{B}=-\frac{1}{N}\sum_{i=1}^{N}\left[w_{p}\cdot y_{i}\cdot \log\sigma \left({\hat{y}}_{i}\right)+w_{n}\cdot \left(1-y_{i}\right)\cdot \log\left(1-\sigma \left({\hat{y}}_{i}\right)\right)\right]$$

#### 2.3.2. Knowledge Distillation Loss

(23) $$L_{K}=\frac{1}{\left|M\right|}\sum_{i\in M}\left[-\sigma \left({\hat{y}}_{i}^{t}\right)\log\sigma \left({\hat{y}}_{i}^{s}\right)-\left(1-\sigma \left({\hat{y}}_{i}^{t}\right)\right)\log\left(1-\sigma \left({\hat{y}}_{i}^{s}\right)\right)\right]$$

where λ is the distillation weight.

The overall loss function is defined as follows:

(24) $$L=L_{B}+\lambda \cdot L_{K}$$

#### 2.3.3. Implementation Details

Our model was developed using Python 3.8, PyTorch Geometric 2.0.4, and PyTorch 1.9.1 + cu111. In our experiments, the hidden layer size of each GCN encoder within the multi-omics encoder architecture was set to 96, while the second hidden layer was configured to 256. The classifier comprised six GCN layers, each with a hidden dimensionality of 200, resulting in a final output dimension of 1. The base training period was established at 900 epochs, with AdamW selected as the model optimizer. The learning rate was configured at 0.00055, the weight decay was set to $8\times 10^{-5}$, and the dropout rate was maintained at 0.2. We utilized six models for integration, with an integration temperature of 0.08, and the training process was enhanced through mixed precision training (AMP).

## 3. Results

### 3.1. Performance Evaluation of the M-GNN

To assess the performance of M-GNN in identifying cancer driver genes, we compared it with five baseline models, EMOGI [11], HGDC [13], SGCD [14], deepCDG [16], and IMVRL-GCN [17], across three PPI networks (STRINGdb [25], CPDB [26], and IRefIndex [27]). We performed 10 independent repetitions of 5-fold cross-validation, each with a different random seed, to assess the stability of our model and to derive the final average results. To ensure fairness, we utilized the same PPI network and multi-omics features for all models, maintaining consistent parameters for the baseline models, as specified in their original publications. In the quintuple-fold cross-validation, we randomly divided the dataset into a training set (75%) and a test set (25%). We evaluated model performance using two metrics: the area under the receiver operating characteristic curve (AUC) and the area under the precision–recall curve (AUPRC).

Table 1 compares the performance of M-GNN and other baseline models across all networks. The experimental results indicate that M-GNN consistently achieves the highest or second-highest AUPR when evaluated on a pan-cancer dataset comprising three PPI networks. On the STRINGdb network, the mean AUPR across the 10 independent runs was 0.8232 with a standard deviation of only 0.0124 (as shown in Supplementary Figure S2), demonstrating the robustness of our model to random variations in data partitioning and negative sample selection. To assess the robustness of M-GNN to the choice of negative sampling ratio, we performed additional experiments on the STRINGdb network by varying the ratio of negative to positive samples in the training set (from 1:1 to using all negatives, i.e., the original ratio of approximately 16:1). As shown in Supplementary Table S5, the mean AUPR remained stable (ranging from 0.8198 to 0.8216), indicating that the model performance was not sensitive to the exact negative sampling strategy. This finding underscores the effectiveness of M-GNN in integrating multi-modal data and effectively synthesizing multi-view data while also demonstrating robust performance, particularly when the number of positive samples is limited.

**Figure 2:**
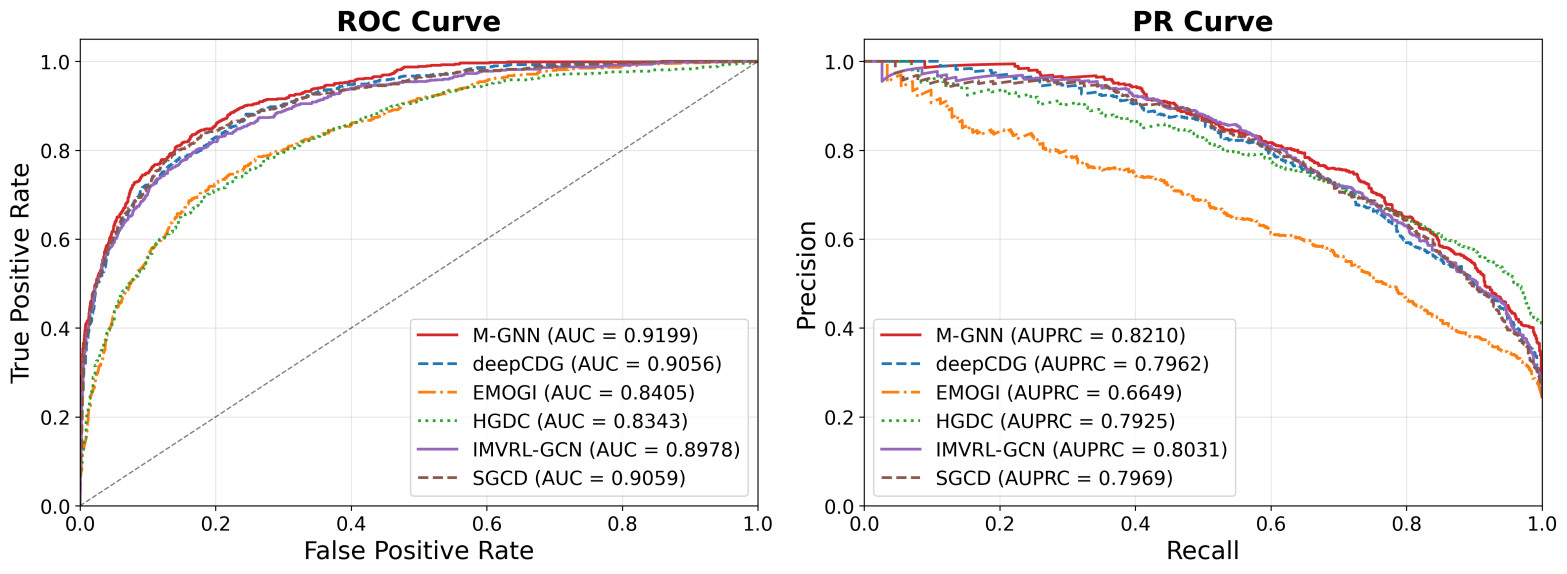
Comparison of ROC curves and PR curves of M-GNN with other baseline models on the STRINGdb network.

Additionally, Figure 2 presents the ROC curve and AUC alongside the precision–recall (PR) curve and AUPRC values for the M-GNN model and various baseline models on the STRINGdb network. The ROC curves show that M-GNN achieves the highest AUC among all models, indicating its strong classification ability in distinguishing between positive and negative samples. Furthermore, when evaluating the model’s performance in scenarios of class imbalance through PR curves, M-GNN also demonstrated strong performance in AUPRC metrics. All selected baselines operate on exactly the same input data (gene-level multi-omics features and PPI networks) and perform the identical task of gene-level driver gene prediction, ensuring fair comparison. This study benchmarks M-GNN primarily against GNN-based competitors rather than attempting a comprehensive comparison across the full landscape of driver gene prediction strategies. Non-GNN methods were excluded because they either require different data formats or cannot leverage network topology, making them unsuitable for evaluating our graph-based architecture. Hyperparameters for each baseline were tuned following their original papers and code using grid search on validation folds to reproduce reported performance. This ensured that the observed improvements were attributable to methodological innovations rather than suboptimal configuration.

### 3.2. Ablation Study

To investigate the significance of various components of M-GNN, we conducted a series of ablation experiments. M-GNN is a multi-omics integration method based on independent GCN encoding. First, we aimed to assess the importance of distinct omics data types; specifically, $X_{M}$ denotes mutation features, $X_{E}$ indicates expression features, $X_{T}$ represents methylation features, and $X_{C}$ corresponds to copy number variation features. Second, we sought to evaluate the importance of different M-GNN module components, along with the significance of additional features, as detailed below:

- $X_{M}+X_{T}$: Represents the use of mutation and methylation signatures as inputs;
- $X_{M}+X_{E}+X_{T}$: Removal of the copy number variation feature;
- $X_{M}+X_{E}+X_{C}$: Removal of methylation features;
- $X_{E}+X_{T}+X_{C}$: Removal of the mutation feature;
- Without independent encoding: Use of a shared encoder to replace the original independent encoder, specifically, using the SAGEConv layer for neighbor sampling and aggregation;
- Independent encoding by GAT: Use of Graph Attention Network (GAT) as the encoder for multi-head attention to capture neighbor importance in different aspects;
- Without attention layer: Fusion of different omics types using simple summation;
- Predict by GCN: Use of only GCNs as a predictor;
- Without structural features: Removal of structural features;
- Without Node2Vec embedding: Removal of Node2Vec embedding features;
- Without teacher model: Removal of the additional teacher model;
- Without feature enhancement: Removal of all additional features.

Table 2 presents the performance changes of the M-GNN following the integration of various components, evaluated through 10 quintuple cross-validations on STRINGdb. First, when comparing the model’s performance after incorporating different omics types, it was found that the performance when only two or three omics were used as inputs was lower than the performance when all the omics were integrated. This finding indicates that distinct omics types contribute uniquely to the model’s efficacy. Second, our validation results underscore the significance of additional features, revealing a marked decline in performance upon the removal of PPI structural features, Node2Vec embeddings, and teacher models. This suggests that incorporating diverse features from multiple sources is effective for performance enhancement. To further dissect the contribution of knowledge distillation, we evaluated two additional variants: using only the teacher logits as input features (without distillation loss) and using only the distillation loss (without teacher features). Both individual components improve performance over the no-teacher baseline, confirming that feature enrichment and loss regularization provide synergistic benefits.

Finally, we considered the significance of various components within the model. We employed GraphSAGE to directly process the entire feature vector, as opposed to the independent encoding of different omics types utilized in the GNN multi-encoder, which demonstrated significantly lower performance. This approach confirmed that distinct omics types necessitate specific feature transformations. Furthermore, independent encoders are more adept at capturing omics-specific patterns, while multi-omics diversity offers unique and crucial biological insights. The effective fusion across multiple omics types can be further augmented through the interaction and integration facilitated by attention layers. Additionally, we observed that the combination of an MLP with a GNN dual-path predictor design was essential, as it surpassed the performance of individual GNN predictions.

### 3.3. Prediction of Potential Cancer Driver Genes

After training M-GNN separately on each PPI network (STRINGdb, CPDB, IRefIndex), we saved the trained models and then used them to predict potential driver genes. The top 100 high-confidence candidate genes were output on each dataset separately, which were subsequently merged and de-emphasized, resulting in a non-redundant set of candidate genes (see Table S2 in the Supplementary Materials). To assess the reliability of the prediction results of the M-GNN model, we compared its predicted potential cancer driver genes with two authoritative literature-derived cancer gene datasets: the CancerMine [42] (a resource of cancer drivers, oncogenes, and tumor suppressors identified through text mining) and the NCG [43] (Network of Cancer Genes) (a database that systematically collects driver genes that are recurrently mutated in a wide range of cancers, including both classical and candidate driver genes).

A total of 201 potential cancer driver genes were predicted by the M-GNN model with high confidence. We found that a total of 182 genes (91.0%) matched at least one record in the database. This high overlap rate strongly suggests that the predictions of M-GNN are highly consistent with the existing knowledge of cancer biology.

Further analysis revealed that 164 (81.6%) of the validated genes were included in the CancerMine database, indicating that their roles as drivers have been extensively documented in the literature. Additionally, 107 (53.3%) of these genes were found in the NCG database, confirming that their functions as drivers across multiple cancer types have been systematically validated. Notably, 88 (48.4%) of the genes were present in both CancerMine and the NCG. These genes, including NOTCH3, PTK2, and CDH2, are recognized as classical cancer drivers that have been substantiated through both literature mining and systematic analysis, thereby reinforcing the reliability of the M-GNN model’s screening capability.

**Figure 3:**
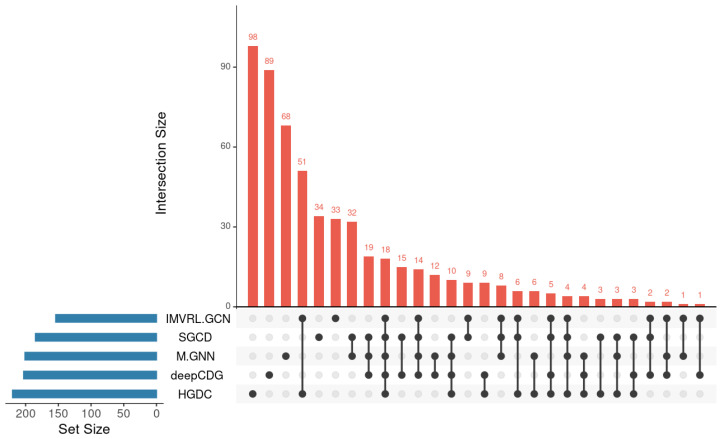
UpSet plot showing the overlap of cancer driver genes identified by M-GNN and other methods. The horizontal bar chart (**left**) represents the “Set Size”, indicating the total number of predicted cancer driver genes for each method. The vertical bar chart (**top**) represents the “Intersection Size”, showing the number of genes shared among specific combinations of methods. Dots connected by lines below the bars indicate which methods are included in each intersection. This visualization highlights both common and unique gene predictions across different models.

We also assessed the overlap of cancer driver genes predicted by M-GNN with those identified by other models. As illustrated in Figure 3, the UpSet diagram reveals the number of genes shared among different models. Our method identified several unique cancer driver genes that were not detected by alternative approaches, including FLT1, a VEGF receptor that plays a crucial role in angiogenesis and is linked to a variety of cancers. Notably, FLT1 is highly expressed in nearly all solid tumors, such as colorectal cancer, hepatocellular carcinoma, breast cancer, ovarian cancer, and glioblastoma, and is recognized as a driver gene [44]. Increasing evidence suggests that SERPINH1 functions as a “tumor promoter gene” by maintaining the “hardness” of the tumor microenvironment and facilitating metastasis. Furthermore, it may serve as a “non-classical” tumor promoter gene in certain cancers [45].

The substantial overlap with established cancer gene databases strongly underscores the reliability and utility of the M-GNN model in identifying potential cancer driver genes. Furthermore, genes predicted by the model that are absent from these prominent databases (e.g., LCP2, FRAS1, FLNB, WNK3, etc.) may signify novel and underappreciated driver events, offering valuable insights for future investigations into cancer mechanisms and target discovery.

### 3.4. Enrichment Analysis

To gain a comprehensive understanding of the biological functions of cancer driver genes predicted by the M-GNN model, we conducted Gene Ontology (GO) and KEGG pathway enrichment analyses on the top-ranked 201 non-redundant candidate genes identified by M-GNN (as described in Section 3.3). Enrichment significance was assessed using a hypergeometric test, and *p*-values were adjusted for multiple testing using the Benjamini–Hochberg false discovery rate (FDR) procedure. Terms and pathways with adjusted FDR < 0.05 were considered significantly enriched. The complete list of all significantly enriched GO terms and KEGG pathways (FDR < 0.05) is provided in Supplementary Table S3. The GO analysis encompassed three ontology categories: Biological Process (BP), Cellular Component (CC), and Molecular Function (MF). In contrast, the KEGG analysis concentrated on signaling pathways and disease-related pathways.

**Figure 4:**
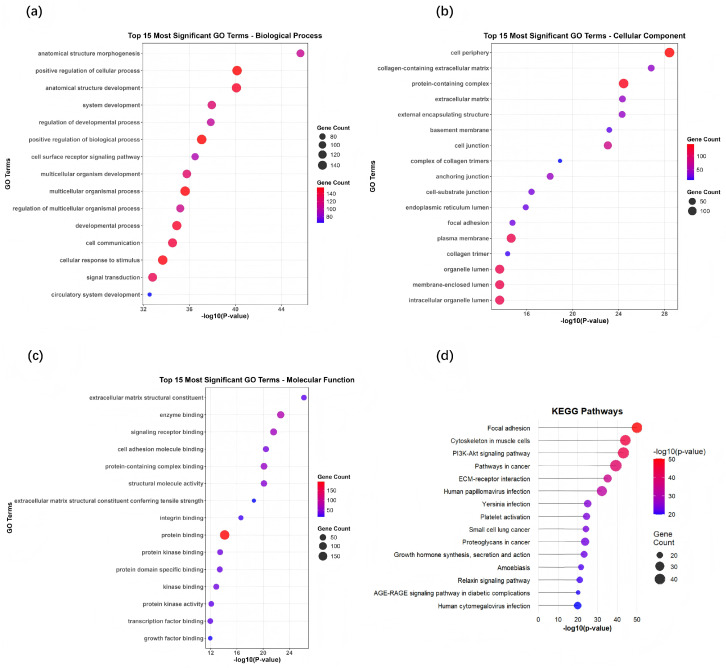
GO and KEGG enrichment analysis of top-ranked predicted cancer driver genes (*n* = 201). (**a**,**b**) show GO Biological Process and Cellular Component; (**c**,**d**) show GO Molecular Function and KEGG pathways. Bubble size represents gene count, color indicates −log10(*p*-value) for GO terms and −log10(FDR) for KEGG. All displayed terms and pathways are significant at Benjamini–Hochberg FDR <0.05.

The results of the GO and KEGG pathway enrichment analyses are presented in Figure 4. These findings indicate that the cancer driver genes predicted by the M-GNN model participate in several key biological processes associated with tumors. All reported terms and pathways passed the significance threshold of FDR < 0.05. The significant enrichment of “anatomical morphogenesis” (FDR = 2.3 × 10^−5^) underscores the crucial role of these genes in tumor tissue remodeling, as well as in invasion and metastasis, which are closely linked to the alteration of the tumor microenvironment. In the analysis of cellular fractions, the enrichment of “collagen-containing extracellular matrix” (FDR = 1.8 × 10^−6^) emphasizes the essential function of the extracellular matrix in tumor progression. Research has demonstrated that collagen remodeling facilitates tumor cell migration and immune evasion [46].

Regarding molecular function, the enrichment of “enzyme-binding” activity (FDR = 3.2 × 10^−4^) implies that these genes are involved in regulating a diverse array of signal transduction networks, particularly those related to protein kinase pathways [47]. KEGG pathway analysis further corroborated that the significant enrichment of the “PI3K-Akt signaling pathway” (FDR = 4.5 × 10^−8^) underscores the pivotal role of the predicted genes in regulating cell survival, proliferation, and metabolism. Abnormalities within this pathway are characteristic of numerous cancers [48].

Concurrently, the enrichment of the “focal adhesion” pathway (FDR = 2.1 × 10^−6^) highlights the critical nature of cell–matrix interactions in tumor invasion and offers valuable insights into the mechanisms underlying cancer metastasis [49].

To assess the stability of key pathway enrichment, we repeated the KEGG analysis on the top 150 and 200 predicted genes using DAVID [50]. The PI3K-Akt signaling pathway (top150: FDR = 1.50 × 10^−24^; top200: FDR = 3.40 × 10^−22^) and focal adhesion (top150: FDR = 7.07 × 10^−27^; top200: FDR = 5.32 × 10^−29^) remained significantly enriched (FDR < 0.05) across both subsets, indicating the robustness of our findings. The full results of the stability analysis are provided in Supplementary Table S4.

We further examined whether the pathway enrichment observed in the predicted driver genes merely reflects the composition of the training positive set by performing the same enrichment analysis on the set of known driver genes used for model training (STRINGdb: 783 genes). As shown in Supplementary Table S6, both the predicted genes and the known driver genes are significantly enriched in classic cancer pathways such as PI3K-Akt signaling (predicted: FDR = 1.04 × 10^−20^; known: FDR = 2.68 × 10^−31^) and focal adhesion (predicted: FDR = 1.55 × 10^−27^; known: FDR = 3.80 × 10^−15^). However, the predicted genes exhibit additional enrichment in processes related to anatomical structure morphogenesis, regulation of cellular processes, and cell surface receptor signaling (FDR < 0.05), which are not significantly enriched in the known driver gene set. These results indicate that, while M-GNN successfully captures the functional signatures of established driver genes, it also identifies novel candidate genes with distinct biological roles, thereby validating the biological plausibility of the model’s predictions.

### 3.5. Drug Sensitivity Analysis

**Figure 5:**
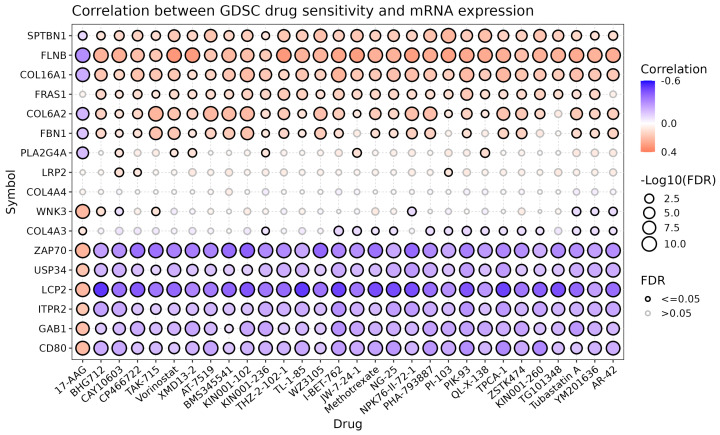
Correlation between drug sensitivity and mRNA expression for 17 selected novel candidate genes. The heatmap shows the top 30 gene–drug associations with significant Spearman correlation (FDR < 0.05). Red indicates positive correlation (higher expression, higher sensitivity), blue indicates negative correlation (higher expression, resistance).

To investigate the potential therapeutic relevance of the predicted driver genes, we selected 17 high-confidence candidate genes that were absent from both the CancerMine and NCG databases (representing potentially novel driver genes) for drug sensitivity analysis. Using the Gene Set Cancer Analysis (GSCA) platform [51] integrated with the GDSC database [52], we evaluated the correlation between gene expression and drug sensitivity. After Benjamini–Hochberg FDR correction (FDR < 0.05), we identified 1535 significant gene–drug associations across the 17 genes. The number of significant associations per gene ranged from 15 (COL4A4) to 160 (LCP2), with absolute Spearman correlation coefficients of up to 0.516. As shown in Figure 5, several clinically relevant associations were identified. Notably, high expression of CD80 was associated with sensitivity to the TAK1 inhibitor NG25 (r = 0.41, FDR = 1.8 × 10^−4^), which has been shown to induce apoptosis in multiple myeloma and breast cancer cells by blocking NF-κB and p38 signaling pathways [53]. Additionally, FBN1 expression correlated with the PI3Kδ inhibitor CAL-101 (Idelalisib) (r = −0.48, FDR = 0.01), a first-in-class PI3K inhibitor approved for the treatment of B-cell malignancies [54]. These findings suggest that even understudied genes predicted by M-GNN may harbor therapeutic potential, providing new insights for targeted therapy development.

## 4. Discussion

In this study, we proposed M-GNN, a multi-modal GCN that integrates multi-omics data with PPI networks for cancer driver gene prediction. The model’s multi-level design—comprising separate omics encoding, attention-based fusion, feature enhancement, and dual-path classification—enables comprehensive integration of heterogeneous data and network topology. Experimental results on three pan-cancer datasets demonstrate that M-GNN achieves competitive performance and outperforms most existing baseline methods, with ablation studies confirming the contribution of each module, particularly the feature enhancement strategy (including structural features, Node2Vec embeddings, and knowledge distillation). Enrichment and drug sensitivity analyses further validated the biological relevance of the predicted genes, linking them to key cancer pathways and potential therapeutic targets.

We acknowledge that ranking-based metrics such as precision@k and NDCG are directly relevant for the practical prioritization of candidate driver genes. Their absence in this study was a deliberate choice grounded in three considerations: First, AUC and AUPRC remain the de facto standards in the field; recent state-of-the-art methods including CancerGATE [55] and MLGCN-Driver [56] rely on these metrics, reflecting the community’s consensus that they effectively capture performance under the severe class imbalance inherent to driver gene prediction. Second, the five baseline models we compared against (EMOGI, HGDC, SGCD, deepCDG, and IMVRL-GCN) were originally evaluated only with AUC and AUPRC, and their raw per-gene prediction scores are not available, making a fair ranking-based comparison infeasible without re-implementing and retraining each baseline—a process that would introduce new sources of variability and could compromise comparability. Third, the repeated cross-validation experiments (Supplementary Figure S2) already demonstrated the stability of our model, and the consistent superiority in AUC and AUPRC across most networks provides strong evidence of its effectiveness. The ablation study (Table 2) further confirms the contribution of each module. Consequently, while ranking metrics would offer additional practical insight, their omission does not undermine the validity of our core conclusions.

Beyond the current application to genomics, epigenomics, and transcriptomics, the proposed M-GNN framework is, in principle, adaptable to other omics modalities such as metabolomics or lipidomics. The core architecture—separate encoders for each modality, attention-based fusion, and topological feature enhancement—does not rely on specific data types and can accommodate any omics data that can be represented as node features in a biological network. However, limitations exist: metabolomics and lipidomics data are often sparser and subject to higher technical variability, requiring dedicated preprocessing; reliable interaction networks for these modalities are less established than protein–protein interaction networks; and the static network assumption may not capture the dynamic nature of metabolic profiles. Despite these challenges, M-GNN provides a flexible foundation for extension to these domains with appropriate network definitions and data preprocessing.

Despite these advances, the current framework relies on static PPI networks and gene-level features, which may not capture dynamic or isoform-specific interactions. Future work will address these limitations by incorporating temporal network data and single-cell sequencing, by exploring transfer learning across cancer types, and by incorporating ranking-based metrics as complementary evaluation measures to enhance generalization and clinical applicability. Additionally, while our sensitivity analyses (Supplementary Figure S2 and Table S5) confirmed the model’s robustness under different negative sampling settings, the fundamental risk that some undiscovered driver genes may still reside in the negative set remains. Therefore, these results should be interpreted with this inherent labeling uncertainty in mind, and future work could explore more reliable negative sample definitions to further strengthen the evaluation.

## 5. Conclusions

We presented M-GNN, a topology-enhanced multi-modal GCN for cancer driver gene prediction. The model achieves robust performance by adaptively fusing multi-omics features, enriching representations with structural and distilled knowledge, and combining GCN and MLP classifiers. The primary innovations of this study are as follows:

- **Multi-level data fusion architecture:** Systematic integration of four omics datasets with PPI networks, fully utilizing complementary information.
- **Adaptive feature learning mechanism:** Attention-based dynamic weighting of different omics for data-driven fusion.
- **Graph structure enhancement strategy:** Incorporation of Node2Vec and structural features to capture functional associations in biological networks.
- **Knowledge distillation technique:** Use of soft labels from a pre-trained teacher model to improve student model performance and stability.

M-GNN provides an efficient computational tool for systematic cancer driver gene discovery and establishes a methodological foundation for integrating multi-modal biomedical data.

## Figures and Tables

**Table 1:** Comparison of AUPRC values between M-GNN and other baseline models across three PPI networks.

Dataset	M-GNN	deepCDG	SGCD	HGDC	IMVRL-GCN	EMOGI
STRINGdb	**0.8211**	0.7962	0.7968	0.7925	0.8030	0.6649
CPDB	0.8007	0.7980	**0.8014**	0.7848	0.7925	0.7019
IRefIndex	**0.7610**	0.7505	0.7608	0.7166	0.7297	0.6567

**Table 2:** Ablation study on STRINGdb (10 × 5-fold cross-validation).

Data or Models	AUC	AUPRC
$X_{M}+X_{T}$	0.9181	0.8109
$X_{M}+X_{E}+X_{T}$	0.9202	0.8208
$X_{M}+X_{E}+X_{C}$	0.9080	0.7884
$X_{E}+X_{T}+X_{C}$	0.9201	0.8203
$X_{M}+X_{E}+X_{T}+X_{C}$	**0.9213**	**0.8215**
Without independent encoding	0.9149	0.8039
Independent encoding by GAT	0.9063	0.8015
Without attention layer	0.8885	0.7926
Predict by GCN	0.9193	0.8170
Without structural features	0.9159	0.8135
Without Node2Vec embedding	0.9113	0.8041
Without teacher model	0.9022	0.7881
Teacher logits only	0.9035	0.7935
Distillation loss only	0.9107	0.8004
Without feature enhancement	0.8969	0.7788

## Data Availability

The source code is available on GitHub at https://github.com/qinqlulu/M-GNN, accessed on 6 January 2026.

## Supplementary Materials

The following supporting information can be downloaded at https://www.mdpi.com/article/10.3390/metabo16040268/s1: Figure S1: The process of generating additional features; Table S1: Information of PPI networks; Figure S2: Mean AUPR and variability for M-GNN across 10 independent runs on the STRINGdb network; Table S2: Predicted Potential Cancer Driver Genes; Table S3: All_significant_pathways; Table S4: Stability analysis of key KEGG pathways across different numbers of top-ranked predicted genes; Table S5: Sensitivity to negative-to-positive sampling ratio on STRINGdb; Table S6: The same enrichment analysis on the set of known driver genes used for model training.
