# Supplementary material for: M-GNN: A Topology-Enhanced Multi-Modal Graph Neural Network for Cancer Driver Gene Prediction
Source: Metabolites. 2026 Apr 16;16(4):268. doi: 10.3390/metabo16040268 (PMC13117570; doi:10.3390/metabo16040268)
Supplement: Supplementary file 1 [file metabolites-16-00268-s001.zip › Supplementary_Material_for___M_GNN_A_Topology_enhanced_Multi_modal_Graph_Neural_Network_for_Cancer_Driver_Gene_Prediction__.pdf]

Supplementary Material for ‘’M-GNN—A Topology-enhanced Multi-modal Graph Neural Network for Cancer Driver Gene Prediction”

Lu Qin[1,4], Wen Zhu[2*], Xinyi Liao[3], and Yujing Zhang[1,4]

[1]School of Mathematics and Statistics, Hainan Normal University, Hainan Haikou 571158, China
[2]School of Mathematics and Systems Science, Guangdong Polytechnic Normal University, Guangzhou 510665, China
[3]China Unicom (Hainan) Industrial Internet Co., Ltd, Hainan Haikou 571924, China
[4]Key Laboratory of Data Science and Intelligence Education, Hainan Normal University, Ministry of Education, Hainan Haikou 571158, China

S1 Information of PPI networks

Table S1: Information of PPI networks

Name	Number of Edges	Number of Nodes	Number of Positive Samples	Number of Negative Samples	Homophily Ratio
CPDB	252,189	13,627	796	2,187	0.1568
STRINGdb	336,549	13,179	783	2,415	0.1889
IRefIndex	371,568	17,013	836	4,056	0.1678

S2 Multi-omics Feature Data

S2.1 Acquisition of Pan-cancer Multi-omics Data

We collect cancer genomics (mutations and copy number), epigenomics (DNA methy lation), and transcriptomics (gene expression) from the cancer genome atlas (TCGA, `https://portal.gdc.cancer.gov/`), encompassing over 29,446 samples across 16 dis tinct cancer types, For each gene, we calculate gene mutation rate, copy number aberrations (CNAs), differential DNA methylation rate, and differential gene expression rate across the 16 cancer types.

S2.2 Preprocessing of Multi-omics Features

The following preprocessing steps were applied to the multi-omics features:

S2.2.1 Gene Mutation Rate

The mutation rate of a gene in a given cancer type is defined as the number of non-silent mutations in that gene divided by its exonic length. The gene mutation rate is calculated as:

$$mf_i^c = \frac{1}{|pc|} \sum_{p \in pc} F_{p,i} \tag{1}$$

For cancer type c, pc represents the set of patients, and $F_{p,i}$ denotes the mutation frequency for gene i in the sample from patient p.

S2.2.2 CNAs

Gene-associated CNAs are collected from TCGA data, encompassing both amplifications and deletions, while ultramutated samples from syn1729383 are excluded from our study. The copy number rate for each gene is defined as the total number of times that gene is either amplified or deleted in a specific cohort.

S2.2.3 Differential DNA Methylation Rate

DNA methylation data were obtained from the Illumina Human Methylation 450K BeadChip for tumor samples and corresponding tumor-adjacent normal tissue samples. The differential DNA methylation rate is calculated as:

$$dm_i^c = \frac{1}{N_d} \sum_{p \in pc} (\beta_{p,i}^t - \beta_{p,i}^n) \tag{2}$$

where $\beta_{p,i}^t$ and $\beta_{p,i}^n$ represent the DNA methylation levels for the tumor sample and the normal sample, respectively, from patient p for gene i in cancer type c. N_d is the number of patients with paired tumor and normal samples.

S2.2.4 Differential Gene Expression Rate

Genes with zero values in more than 10% of the total samples were filtered out to reduce noise. Subsequently, all data were log2-transformed. The differential gene expression rate is calculated as:

$$ge_i^c = \frac{1}{N_e} \sum_{p \in pc} \log_2 \left(\frac{V_{p,i}^t}{V_{p,i}^n} \right) \tag{3}$$

where $V_{p,i}^t$ and $V_{p,i}^n$ represent the gene expression levels for the tumor sample and the normal sample, respectively, from patient p for gene i in cancer type c. N_e is the number of patients with both tumor and normal samples in the gene expression data.

By concatenating these vectors across all cancer types, a 64-dimensional feature vector is obtained for each gene.

S2.2.5 Handling Missing Values

Missing feature values were imputed using the median value of the same gene across all samples.

S2.2.6 Normalization

Each feature was normalized to have a mean of 0 and a standard deviation of 1 using the formula:

$$x' = \frac{x - \mu}{\sigma} \tag{4}$$

where μ is the mean of the feature and σ is the standard deviation.

S3 Additional Feature Acquisition

As illustrated in Figure S1, we incorporated additional features, including PPI structural features, Node2Vec embeddings, and outputs from the teacher model. For the structural features, we computed seven metrics for each node, which included node degree (Newman, 2003; Kipf & Welling, 2017) [1] [2] [3], the standard deviation of neighbor degree (Pastor-Satorras et al., 2001) [4], and personalized PageRank (Page et al., 1999) [5]. Additionally, we derived 7-dimensional multiscale structural features dynamically for various

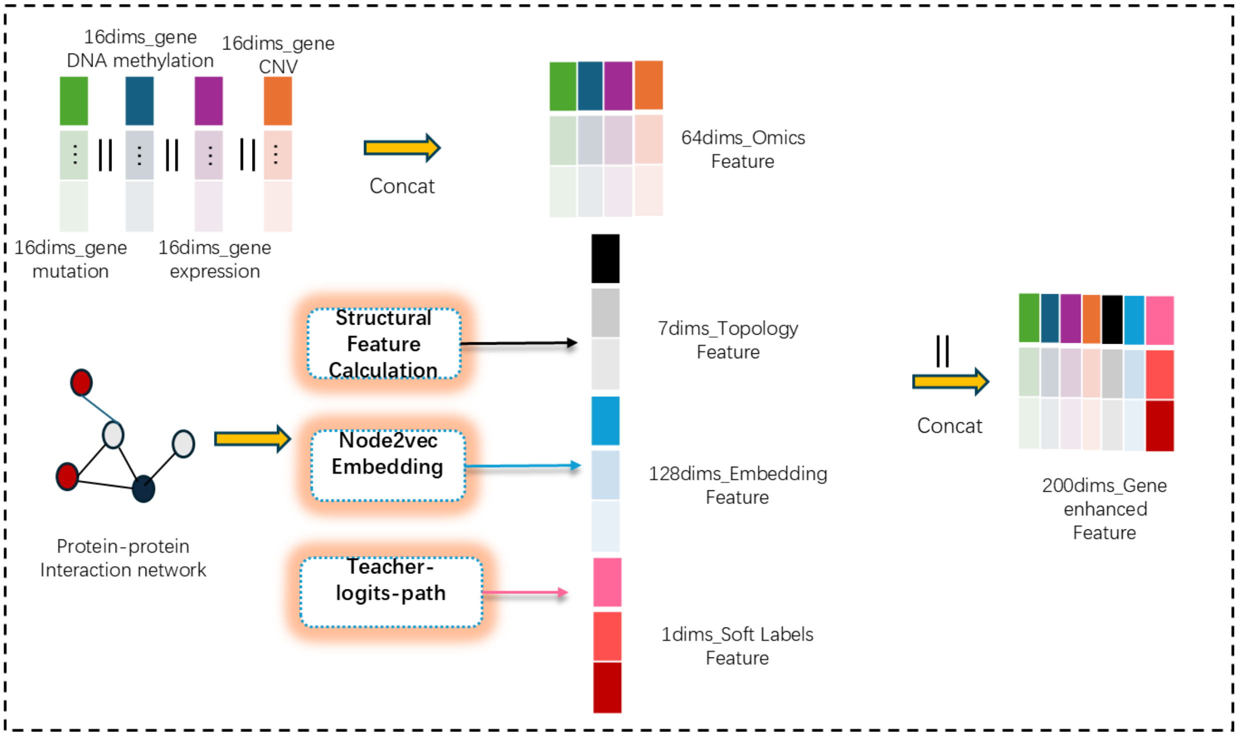

Figure S1: The process of generating additional features."||" or "Concat" for splicing.

input graphs. We employ the Node2Vec algorithm (Grover & Leskovec, 2016) [6] to derive distributed embedding representations of nodes. This algorithm generates node sequences through biased second-order random walks and applies the Skip-gram model (Mikolov et al., 2013) [7] to optimize the co-occurrence probability of a node with its contextual nodes.

We utilize the knowledge distillation framework (Hinton et al., 2015) [8] to improve model performance. Initially, we independently train the model on the STRINGdb dataset, enhancing its configuration with increased capacity, extended training duration, and a more sophisticated integration approach. Following training completion, the teacher model produces predicted logits for all training nodes, which are stored as files to guideudent training through feature splicing and distillation loss. The detailed description is as follows:

S3.1 Basic Degree Features

Four basic degree features were extracted: node degree, log degree, square root degree, and inverse square root degree. These features reflect the direct connectivity scale of nodes and are transformed to mitigate degree distribution skewness (referencing Barabási and Albert, 1999 [1]; Leskovec and Faloutsos, 2006 [2]; Kipf and Welling, 2017 [3]).

Node Degree In network science, degree is the most fundamental centrality measure, indicating the number of direct connections of a node.

$$d_i = \sum_{j=1}^{n} A_{ij} \tag{5}$$

where A is the adjacency matrix.

Log Degree

$$\log\_\deg_i = \log(1 + d_i) \tag{6}$$

Motivation: Logarithmic transformation is commonly used to handle heavy-tailed distributions, making the data closer to a normal distribution. Using log degree in graph neural networks can mitigate the influence of high-degree nodes.

Square Root Degree

$$\sqrt{\deg}_i = \sqrt{d_i + 1} \tag{7}$$

Motivation: The square root transformation is a variance-stabilizing transformation often used for Poisson-distributed data. In graph structures, it serves as a mild normalization for degree.

Inverse Square Root Degree

$$\text{inv\_}\sqrt{\deg}_i = \frac{1}{\sqrt{d_i + \epsilon}} \tag{8}$$

Motivation: The inverse square root degree is commonly used for symmetric normalization in graph convolution, as mentioned earlier. It helps reduce the weight of high-degree nodes, preventing feature vectors from being dominated by a few highly connected nodes.

S3.2 Neighbor Statistical Features

Two neighbor statistical features were computed: mean neighbor degree and standard deviation of neighbor degree. These features capture the local connectivity patterns of nodes (referencing Newman, 2002 [4]).

S3.2.1 Mean Neighbor Degree

$$k_{nn}(i) = \frac{1}{d_i} \sum_{j \in \mathcal{N}(i)} d_j \tag{9}$$

where $\mathcal{N}(i)$ is the set of neighbors of node i.

Theoretical Significance

- Basis for assortativity coefficient: Measures whether nodes tend to connect to nodes with similar degrees.
- Identifies hierarchy in networks:
  - $k_{nn}(i) > d_i$: Node connects to more central neighbors.
  - $k_{nn}(i) < d_i$: Node connects to less central neighbors.

S3.2.2 Standard Deviation of Neighbor Degree

$$\sigma_{nn}(i) = \sqrt{\frac{1}{d_i} \sum_{j \in \mathcal{N}(i)} (d_j - k_{nn}(i))^2} \tag{10}$$

Motivation: The standard deviation of neighbor degree measures the dispersion of degrees among a node's neighbors, reflecting the consistency of its connections. A small standard deviation indicates that the node is connected to neighbors with similar degrees, while a large standard deviation indicates connections to neighbors with varied degrees.

S3.3 Global Influence Feature

One global influence feature was included: Personalized PageRank score, which measures the global importance of a node within the network (referencing Brin and Page, 1998; Haveliwala, 2002 [5]).

S3.3.1 Personalized PageRank

$$\mathbf{pr}^{(t+1)} = (1-\alpha) \cdot \mathbf{M} \cdot \mathbf{pr}^{(t)} + \alpha \cdot \mathbf{v} \tag{11}$$

where $\mathbf{M}$ is the transition matrix (column-stochastic), $\mathbf{v}$ is the personalization vector (here using a uniform distribution, i.e., $\mathbf{v} = \frac{1}{n}\mathbf{1}$), and α is the damping factor.

Motivation: Measures the global importance of a node within the network; combines local connectivity and random walk information; particularly effective in protein-protein interaction networks, as important proteins often form densely connected modules.

By integrating these features, the topological attributes of nodes can be described at different scales (local, mesoscopic, global).

S3.4 Node2Vec Embedding Features

To capture functional similarity and global structural information of genes within the PPI network, We employ the Node2Vec algorithm (Grover & Leskovec, 2016) [6] to derive distributed embedding representations of nodes. This algorithm generates node sequences through biased second-order random walks and applies the Skip-gram model (Mikolov et al., 2013) [7] to optimize the co-occurrence probability of a node with its contextual nodes. Specifically, for the protein interaction network $G = (V, E)$, we produce multiple random walk sequences for each node. The wandering strategy is governed by the parameters p and q, where p modulates the inclination to return to the starting node, and q dictates the propensity to explore new directions. The resulting embedding vectors effectively capture the structural roles and functional similarities of the nodes within the network. The specific parameters are as follows:

- Walk length: 60
- Walks per node: 8
- Return parameter (p): 0.8
- In-out parameter (q): 1.2
- Embedding dimension: 128
- Context size: 10
- Epochs: 4

The generated embedding vectors were normalized and used as additional 128-dimensional features.

S3.5 Teacher Model

The teacher model, trained independently on the full training set with an enhanced configuration, was used to generate soft-label predictions for knowledge distillation. The specific parameters are as follows:

- Ensemble members: 10
- Epochs: 1200
- Hidden layer 1 dimension: 128
- Hidden layer 2 dimension: 512
- Dropout rate: 0.15

- Learning rate: 0.0003
- Weight decay: 5e-5
- Early stopping patience: 200
- Class balance parameter (cb_beta): 0.998
- Evaluation interval: 25
- Use exponential moving average (use_ema): True
- EMA decay rate: 0.995

The student model was trained using knowledge distillation guided by the teacher model's soft labels. The key hyperparameters, optimized on the validation set, are listed below:

- Learning rate: 0.00055
- Hidden layer dimensions: 96 and 256
- Training epochs: 900
- Number of ensemble members: 6
- Distillation temperature: 0.08
- Distillation loss weight: 0.5 (tuned on validation set)

S4 List of Predicted Potential Cancer Driver Genes

Table S2: Predicted Potential Cancer Driver Genes

ABCA1	ACTB	AHNAK	AKAP13
ANK3	APOB	APP	ARHGAP32
ATF2	ATF3	BMPR2	CD80
CDH2	CDON	CFTR	CHD9
CHUK	COL11A1	COL11A2	COL16A1
COL1A2	COL4A1	COL4A2	COL4A3
COL4A4	COL4A5	COL4A6	COL5A1
COL5A2	COL5A3	COL6A2	COL6A3
COL7A1	CRK	CSF2RB	CTNNA1
CYP2A6	DMD	DOCK1	DSP
DST	ELMO1	EPHA2	FADD
FANCM	FBN1	FBN2	FLNB
FLNC	FLT1	FN1	FOS
FRAS1	FREM2	FYN	GAB1
GATA4	GHR	GLI3	GRB2
GRIA2	GRM1	HDAC9	HNF4A
HSPA8	HSPG2	IGF2	IL2RA
IL2RG	IL3RA	INPP5D	INPPL1
INSR	IQGAP1	IRAK1	IRS1
ITGA1	ITGA4	ITGB1	ITGB3

ITGB4	ITPR1	ITPR2	ITPR3
KLB	KMT2B	L1CAM	LAMA1
LAMA3	LAMA4	LAMB1	LAMC1
LAMC2	LCP2	LPL	LRP1
LRP2	LRP6	LRRK2	LYN
MACF1	MAMLD1	MAP2	MAP3K7
MAPK10	MAPK3	MAPT	MED1
MED13	MEF2C	MEIS1	MLH3
MMP3	MYCBP2	MYH10	MYH7
MYLK	NCAM1	NEB	NEDD4
NFATC1	NFKBIA	NGF	NID1
NOS1	NOTCH3	NOTCH4	NPHS1
NR3C1	NR3C2	NRG3	NRIP1
NRP1	NTRK2	PIK3CD	PIK3CG
PIK3R2	PLA2G4A	PLCG2	PLEC
PLG	PPARGC1A	PRKACB	PRKCA
PRKCE	PRKD1	PRKDC	PROX1
PTK2	PTK2B	PTPRJ	PTPRZ1
RASA1	RBPJ	RELN	ROBO1
ROCK1	ROCK2	RUNX2	RXRA
RYR2	SALL1	SATB1	SERPINH1
SHC1	SHH	SLIT2	SLX4
SMARCA2	SMARCC2	SMARCD3	SOS1
SP1	SPI1	SPTA1	SPTAN1
SPTBN1	STAT1	STAT4	STAT5A
SVIL	SYNE1	TCF4	TFAP2A
TGS1	TLE4	TLN1	TNK2
TNRC6A	TP53BP1	TRIB3	TTN
USP34	UTRN	VCAN	VWF
WNK1	WNK3	ZAP70	ZFPM2
ZNF423			

S5 Stability analysis of key KEGG pathways across different numbers of top-ranked predicted genes

Table S4: Stability analysis of key KEGG pathways across different numbers of top-ranked predicted genes.

**Gene set**	**Pathway**	**Count**	**P-value**	**FDR (Benjamini)**
top150	PI3K-Akt signaling pathway	41	1.90e-22	1.50e-24
top150	Focal adhesion	35	1.79e-24	7.07e-27
top200	PI3K-Akt signaling pathway	45	4.01e-24	3.40e-22
top200	Focal adhesion	42	2.10e-31	5.32e-29

S6 Mean AUPR (with standard deviation) for M-GNN across 10 runs on STRINGdb, alongside baseline point estimates

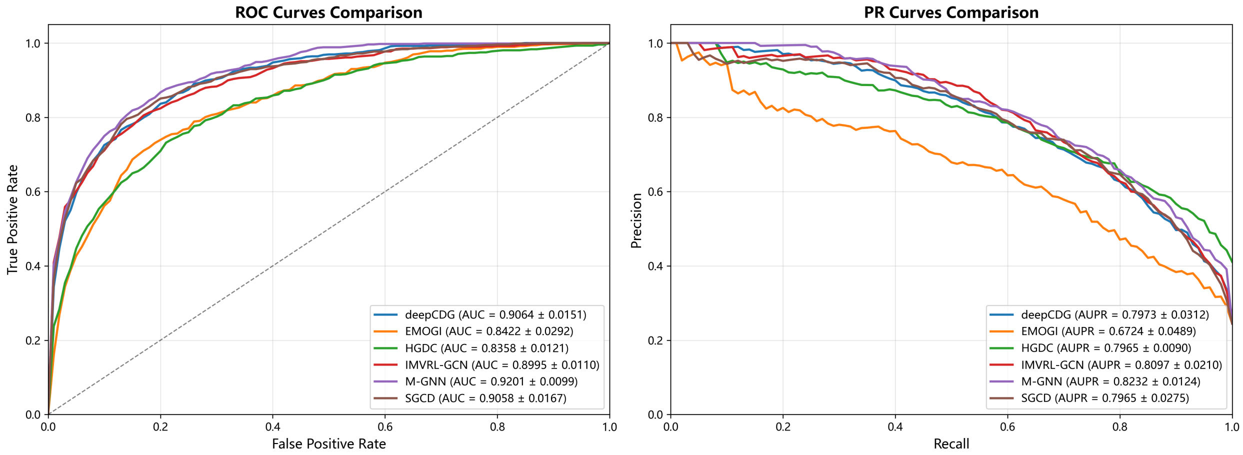

Figure S2: Mean AUPR and variability for M-GNN across 10 independent runs on the STRINGdb network, compared with the point estimates of baseline models. Error bars indicate the standard deviation of the mean.

S7 Evaluate the robustness of model to the negative sampling strategy

Table S5: Sensitivity to negative-to-positive sampling ratio on STRINGdb.

Negative:Positive ratio	Mean AUC	Mean AUPR
1:1	0.9187	0.8198
2:1	0.9193	0.8199
5:1	0.9206	0.8210
10:1	0.9206	0.8195
All negatives (≈16:1)	0.9210	0.8216

References

[1] Barabási, A.-L., Albert, R. Emergence of scaling in random networks. *Science* **286**, 509–512 (1999). DOI: 10.1126/science.286.5439.509

[2] Leskovec, J., Kleinberg, J., Faloutsos, C. Graphs over time: densification laws, shrinking diameters and possible explanations. In *Proceedings of the eleventh ACM SIGKDD international conference on Knowledge discovery in data mining* (KDD '05). Association for Computing Machinery, New York, NY, USA, 177–187 (2005). https://doi.org/10.1145/1081870.1081893

[3] Kipf, T. N., Welling, M. Semi-supervised classification with graph convolutional networks. *arXiv:1609.02907* (2016).

[4] Newman, M. E. J. Assortative mixing in networks. *Physical Review Letters* **89**(20), 208701 (2002). https://doi.org/10.1103/PhysRevLett.89.208701

[5] Haveliwala, T. H. Topic-sensitive PageRank. In *Proceedings of the 11th international conference on World Wide Web* (WWW '02). Association for Computing Machinery, New York, NY, USA, 517–526 (2002). https://doi.org/10.1145/511446.511513

[6] Grover, A., & Leskovec, J. (2016). Node2vec: Scalable Feature Learning for Networks. In *Proceedings of the 22nd ACM SIGKDD International Conference on Knowledge Discovery and Data Mining* (pp. 855–864). https://doi.org/10.1145/2939672.2939754

[7] Mikolov, T., Sutskever, I., Chen, K., Corrado, G. S., & Dean, J. (2013). Distributed representations of words and phrases and their compositionality. In *Advances in Neural Information Processing Systems* (pp. 3111–3119).

[8] Hinton, G. E., Vinyals, O., & Dean, J. (2015). Distilling the Knowledge in a Neural Network. *arXiv preprint*. arXiv:1503.02531.
